# A glance into the future of diagnosis and treatment of spondyloarthritis

**DOI:** 10.1177/1759720X221111611

**Published:** 2022-07-22

**Authors:** Victoria Navarro-Compán, Joerg Ermann, Denis Poddubnyy

**Affiliations:** University Hospital La Paz and IdiPaz, Madrid, Spain; Division of Rheumatology, Inflammation and Immunity, Brigham and Women’s Hospital and Harvard Medical School, Boston, MA, USA; Department of Gastroenterology, Infectiology and Rheumatology (Including Nutrition Medicine), Charité – Universitätsmedizin Berlin, Corporate Member of Freie Universität Berlin and Humboldt-Universität zu Berlin, Hindenburgdamm 30, Berlin 12203, Germany; Epidemiology Unit, German Rheumatism Research Centre, Berlin, Germany

**Keywords:** ankylosing spondylitis, axial spondyloarthritis, diagnosis, treatment

## Abstract

The last two decades have seen major developments in the field of spondyloarthritis (SpA), but there are still important unmet needs to address. In the future, we envisage important advances in the diagnosis and treatment of SpA. In the diagnosis of SpA, the use of online and social media tools will increase awareness of the disease and facilitate the referral of patients to rheumatology clinics. In addition, more specific diagnostic tests will be available, especially advanced imaging methods and new biomarkers. This will allow most patients to be diagnosed at an early stage of the disease. In the treatment of SpA, an increasing number of novel treatment targets can be expected, most of which will be directed against intracellular enzymes. We hope to see more strategy trials shaping treatment pathways in SpA and accommodating principals of precision medicine. Approved treatment options will be available for both axial and peripheral SpA. We also hope to intervene not only at the inflammation level but also at the level of underlying immunological processes that might be associated with a higher probability of long-standing remission if not a cure. Finally, artificial intelligence techniques will allow for the analysis of large-scale data to answer relevant research questions for the diagnosis and management of patients with SpA.

## Introduction

Spondyloarthritis (SpA) is an umbrella term for a group of inflammatory immune-mediated diseases with commonalities in genetic risk factors, disease mechanisms and clinical features such as axial skeletal involvement and a typical pattern of peripheral joint involvement (mono- or oligoarthritis of the lower extremities, enthesitis, dactylitis) as well as extra-musculoskeletal manifestations (psoriasis, acute anterior uveitis, chronic inflammatory bowel disease).^1^ Depending on the leading manifestation, SpA can be classified as axial (axSpA, predominant involvement of sacroiliac joints and spine) or peripheral (pSpA, predominant peripheral arthritis, enthesitis and dactylitis). Ankylosing spondylitis (AS, currently termed radiographic axSpA) is a form of axSpA with already developed structural damage in the sacroiliac joints visible on radiographs, while the term non-radiographic axSpA is used to classify patients without such damage.^2^

In recent decades, several major breakthroughs have substantially improved the diagnosis and treatment of this condition. The introduction of magnetic resonance imaging (MRI) in the diagnostic approach has made early detection of inflammatory changes in the sacroiliac joints and spine, and, therefore, early diagnosis is possible.^3^ Furthermore, new classification criteria covering the entire disease spectrum and a unified nomenclature have been developed,^4^ understanding of the disease mechanisms have been improved,^5^ guidelines to recognize patients with a high probability of SpA at the primary care level have been developed,^6^ the role of imaging in the diagnostic and classification process have been precisely defined,^7,8^ outcome measurements have been refined^9,10^ and the assessment core set has been updated.^11^ The discovery of the role of tumour necrosis factor (TNF) and, subsequently, interleukin-17A (IL-17A) in the pathophysiology of SpA revolutionized the treatment of this condition.^12^

Nevertheless, we are facing challenges and unmet needs in SpA. The diagnostic delay in axSpA is still quite high, with a mean duration between symptom onset and diagnosis of 5–7 years^13,14^ in Europe and possibly even longer in the United States.^15^ These numbers represent certainly an improvement as compared with the diagnostic delay in AS of about 10 years reported two decades ago^16^ but still indicate a clear unmet need. Efforts to shorten this delay are associated with the risk of overdiagnosis, as there are no pathognomonic clinical changes, highly specific lab tests and even imaging findings such as bone marrow oedema in the sacroiliac joints may occur as a reaction to mechanical stress even in healthy subjects and in persons without inflammatory disease.^17–19^ Furthermore, despite the high efficacy of the currently available treatments (non-steroidal anti-inflammatory drugs – NSAIDs, TNF, IL-17A inhibitors and Janus kinase – JAK – inhibitors), there are still patients who do not respond to therapy at all, who have lost their initial response or who do not achieve remission – the ultimate treatment target in this chronic inflammatory disease. We are also still far away from curing the disease or achieving drug-free remission in the majority of patients. In this review, we will attempt to elucidate the future of SpA based on the current unmet needs and currently ongoing promising developments, which might have an impact on the diagnosis and treatment of SpA in the coming years.

## Diagnosis

Currently, one of the most important unmet needs in the field of axSpA (if not the most important) is to shorten the time it takes for patients to be diagnosed, which is on average 5–7 years from symptom onset.^20^ Approximately 50–60% of patients are diagnosed when irreversible structural damage has already occurred.^21,22^ Recent studies have shown that the delay in diagnosis is mainly because of the arduous journey that axSpA patients follow before reaching rheumatology clinics. They are often seen earlier by other specialists, such as orthopaedic surgeons or physiotherapists, that is, healthcare professionals dealing with back pain, and by ophthalmologists, gastroenterologists or dermatologists, that is, specialist managing disorders, which are known to be associated with SpA.^23^ This journey is associated with a lack of awareness of axSpA among primary care physicians and other specialists as well as the general population and with the low ratio of rheumatologists per capita. Multiple referral strategies have been developed.^24,25^ These strategies are not always implemented in clinical practice, however, and the diagnostic delay remains high.^26^

In the future, it is expected that referral strategies will be implemented to identify patients at an early stage of the disease. In this respect, it seems that digital tools can help to refer patients in an optimal way and to spread the knowledge of the disease among different specialists and the general population. Given the shortage of rheumatologists, innovative healthcare models are needed. Implementation of e-consultation programmes may significantly reduce wait times while assuring prioritization of inflammatory diseases and improving communication between healthcare levels.^27^ Another promising strategy is self-referral. Recent data have shown that an online self-referral tool can be used in specialized centres in addition to a physician-based referral strategy to improve early diagnosis and to increase awareness of axSpA, especially in people younger than 40 years old.^28^ In addition, the use of social media and electronic patient portals seems to be useful in distributing self-referral strategies. It might also be possible to identify patients with possible axSpA using electronic medical record data based on patterns of medical problems, prescriptions and utilization of healthcare resources. With the use of these tools and SpA knowledge dissemination programmes, it is expected that in 10 years, the referral time of patients with suspected axSpA to rheumatology practices will decrease substantially.

Once patients with suspected SpA reach rheumatology clinics, there are also difficulties in diagnosing the disease. To date, there is no gold standard diagnostic test, so establishing a diagnosis of SpA is not always straightforward. This is a complex process combining pattern recognition and clinical reasoning.

Conventional radiography of the sacroiliac joints is still the first imaging test recommended in the diagnostic process for patients with predominantly axial disease.^29^ Radiography can only detect irreversible structural damage, however. Another limitation is the large inter-reader variability when interpreting sacroiliac joint radiographs. Data from pivotal studies exploring the use of artificial intelligence in the interpretation of sacroiliac joint radiographs have shown that deep artificial neural networks allow accurate detection of definitive radiographic sacroiliitis relevant to the diagnosis of axSpA and could therefore be used in the future in non-specialized centres to assist during the diagnostic process.^30^ Detection of definite radiographic sacroiliitis would, however, not solve the problem of a large diagnostic delay as structural changes in the sacroiliac joints take months to years to develop. The great advance in diagnostic tools in recent years has undoubtedly been the use of MRI, especially of the sacroiliac joints, which now makes it possible to detect inflammation (bone marrow oedema) without the need for irreversible damage to have occurred. Initially, efforts focused on high sensitivity for an early diagnosis.^29^ More recent studies have focused on specificity in order to avoid overdiagnosis. Much progress has been made in the technical aspects while performing sacroiliac joint MRI in clinical practice. The interpretation of MRI studies in this setting remains challenging, however. Recent studies have shown that it is important to consider specific contexts or other pathologies that may be associated with findings similar to those observed in axSpA.^31^ Among the contexts to take into account, anatomical variation and recent pregnancy are relevant.^32–35^ In addition, other diseases – including osteitis condensans ilii, gout and diffuse idiopathic hyperostosis – should be considered as differential diagnoses of sacroiliitis.^18,36,37^ It is expected that in the future, the implementation of all the lessons learned in studies in healthy populations and other diseases will facilitate the correct interpretation of MRI findings in clinical practice and improve the accuracy of this exam in the decision-making process. Artificial intelligence approaches might also help in interpretation of MRI – several working groups are working in the field and the results are expected soon.

Furthermore, the role of structural lesions detected by different techniques in the diagnosis of axSpA remains to be elucidated. A recent study compared computed tomography (CT) with conventional radiography and MRI of sacroiliac joints^38^ and found that CT had the best accuracy, highlighting the importance of structural lesions for the differential diagnosis in axSpA. Patients included in this study had a mean symptom duration over 6 years, however; therefore, these results need to be confirmed in other cohorts with shorter disease durations. Low-dose CT approaches have been developed that permit three-dimensional (3D) evaluation of the sacroiliac joints with a radiation dose comparable with plain radiography.^39,40^ Moreover, the development of new MRI techniques that support the detection of structural lesions is an active field. A recent study found that susceptibility-weighted imaging (SWI) in patients with axSpA depicts erosions and sclerosis more accurately than standard T1-weighted imaging using CT as a reference standard.^41^ Another promising approach is synthetic CT, also called bone MRI. Here, an algorithm is used to derive CT-like images from MRI raw data.^42^

Currently, conventional radiography is still the first imaging examination recommended for diagnosis. In young patients or patients with a short duration of symptoms, however, radiography may not detect any changes. In the future, MRI may replace sacroiliac radiography as the first exam in the diagnostic process of axSpA. This decision, however, will be influenced by other aspects, such as the costs and the accessibility of MRI, which thus far is rather limited in many centres. When pSpA is suspected, ultrasound or MRI may be used to detect peripheral enthesitis, tenosynovitis, bursitis and arthritis, which may support the diagnosis of SpA.^29^ Nevertheless, only a small number of epidemiological and clinical studies have addressed this clinical entity as a separate disease, and further studies including this specific population should be performed in the future.^43^

Furthermore, the utility of new imaging techniques for the diagnosis of SpA also remains to be explored. Among these, immunoimaging studies can provide very interesting data in the future. Immunoimaging is a developing technology that aims at studying disease using imaging techniques (e.g. positron emission tomography) in combination with radiolabelled immunoglobulin-derived targeting probes.^44^ In a study of patients with rheumatoid arthritis (RA) and SpA, typical joint involvement patterns in peripheral and axial disease were detected using radiolabelled certolizumab pegol.^45^ Here, the use of nanobodies, instead of monoclonal antibodies, may also be an alternative in the future.^46^ In contrast to histological studies, this technique may represent an opportunity to reliably and non-invasively detect inflammation accurately and thus monitor immunological processes.^47^ The application of these new techniques for the diagnosis of SpA is something that will have to be evaluated in the future, however.

Currently used laboratory biomarkers – such as human leucocyte antigen (HLA)-B27 status and acute phase reactants – have, at best, moderate diagnostic value.^48^ Improved biomarkers for axSpA to assist with early diagnosis are needed. Advances in a range of omics technologies that permit profiling of the genome, microbiome, transcriptome, proteome and metabolome have raised hopes that novel and more informative biomarker can be developed. The substantial contribution of non-HLA loci to AS heritability suggests a role for polygenic risk scores in axSpA diagnosis. In addition, serum levels of antibodies against the HLA class II invariant chain (CD74) were increased in patients with axSpA compared with healthy individuals, but this finding has proven challenging to replicate.^49^ Moreover, several studies have observed that patients with AS have a distinct microbiome that could be used to distinguish patients with AS from healthy individuals.^50^ Future developments in the ‘omics’ field will probably involve combinations of biomarkers that require novel statistical approaches to analyse and to produce easy-to-interpret metrics for clinical application. Large data sets are required to establish successful biomarker discovery and validation programmes. In this sense, having an increasingly digitalized society will undoubtedly favour the creation of extensive databases that will enable research studies to be carried out to identify the disease early and accurately. Over the last decades, healthcare institutions have increasingly abandoned clinical records in paper form and have started to store a large amount of medical information in electronic health records.^51^ While some clinical data are codified, however, the great majority of relevant clinical information remains embedded within the unstructured narrative free text. The use of artificial intelligence techniques such as deep learning and natural language processing will help to effectively use large routine care data sets in research.^52,53^

In summary, in the diagnosis of SpA, we can expect in the future the use of online and social media tools to increase awareness of the disease and facilitate the referral of patients to rheumatology clinics at an early stage of the disease. In addition, the performance of diagnostic tests will improve, with a special focus on imaging techniques and new biomarkers. It is also expected that most patients will be diagnosed at an early stage of the disease. Finally, the use of artificial intelligence will allow for the analysis of large data sets to answer relevant research questions.

## Treatment

A number of highly effective anti-inflammatory substances can be applied in the treatment of patients with active axSpA: NSAIDs, which are normally used first, biological disease-modifying antirheumatic drugs – bDMARDs (TNF and IL-17A inhibitors) and targeted synthetic disease-modifying antirheumatic drugs – tsDMARDs (JAK inhibitors), which are used in patients who do not respond to or do not tolerate NSAIDs. Of these, only NSAIDs are recommended for ‘on-demand’ treatment (depending on symptoms); the other drugs should be taken continuously and long-term. The risk for disease relapse upon discontinuation is nearly always higher than 50% (almost 100% in advanced disease, lower in earlier disease and in non-radiographic axSpA^54–56^). Conventional synthetic disease-modifying antirheumatic drugs (csDMARDs) – such as methotrexate or sulphasalazine – no longer play a meaningful role in axSpA.^12^ Systemic steroids should not be used long term; a short-term treatment course might be beneficial^57^ as a ‘bridging’ therapy as a treatment for a disease flare.

Currently (2022), five TNF inhibitors (adalimumab, certolizumab pegol, etanercept, golimumab and infliximab), two IL-17A inhibitors (ixekizumab and secukinumab) and two JAK inhibitors (tofacitinib and upadacitinib) are approved in the European Union (EU), United States and many other countries for the treatment of axSpA (infliximab, tofacitinib and upadacitinib are approved currently for AS only). Additional IL-17 inhibitors (IL-17 receptor antagonist brodalumab, IL-17A inhibitor netakimab and IL-17A/F inhibitor bimekizumab) as well as the JAK-1 inhibitor filgotinib showed efficacy in axSpA but are not yet approved for the indication in the EU and United States. We also expect that nanobodies – a novel class of therapeutic proteins based on single-domain, camelid, heavy-chain-only antibodies – directed against IL-17 (such a sonelokimab blocking IL-17 A/F^58^) or TNF will show efficacy in SpA and will become available as treatment options.

The efficacy of the currently approved b- and tsDMARDs with regard to musculoskeletal disease manifestations of SpA is about the same. There are differences in efficacy against extra-musculoskeletal manifestations, however: for example, IL-17A inhibitors are more effective than TNF inhibitors in psoriasis; monoclonal TNF antibodies (or a PEGylated Fab fragment of the monoclonal antibody such as certolizumab pegol) are preferred in the presence of inflammatory bowel disease or active/recurrent acute anterior uveitis. As of today, there are no accepted parameters that could help rheumatologists decide which b- or tsDMARD should be given as first-line or as second-line therapy in the case of non-response, indicating a lack of predictors of selective response. Treatment guidelines tend, therefore, to refer to ‘usual practice’ taking into account a larger experience with particular drug classes (such as TNF inhibitors) when considering the choice of particular drug classes and their order.^59^ There is an urgent need for strategy clinical trials (including head-to head comparisons of different modalities) in SpA that could provide important information on potential differences in available strategies: specific choice of the first-, second-, third-line; staying within the drug class *versus* switching to another class, possibility of dose escalation *versus* combination of b- and tsDMARDs for non-responders. Furthermore, these trials could serve as a source of biomaterial for the identification of response/non-response predictors^60,61^ as an important step towards precision medicine in SpA. Most likely, the mentioned trials will need to be conducted as investigator-initiated studies given low interest of pharmaceutical industry in this kind of research questions and concerns about potential negative results related to efficacy or safety.

In a few years, we expect generic JAK inhibitors to enter the market, which should result in a substantial reduction in cost. It is, however, unclear whether the cost reduction will change the place of these substances in the treatment algorithm. A recent study comparing the safety of tofacitinib with TNF inhibitors (adalimumab or etanercept) in patients with RA and at least one risk factor for cardiovascular disease failed to demonstrate non-inferiority of tofacitinib in terms of cardiovascular and cancer risk in the studied patient population.^62^ The observed differences were largely attributable to patients above 65 years with multiple risk factors; nonetheless, these results triggered a large ongoing discussion about the safety of JAK inhibitors as a class.

There are several compounds (mostly small molecules), which will probably be investigated for the indication of axSpA in the next couple of years and may become available to rheumatologists within the next 10 years. They include, for example, the tyrosine kinase-2 (TYK-2)/JAK-1 inhibitor brepocitinib [positive phase I data in psoriatic arthritis presented at American College of Rheumatology (ACR) 2021 Convergence congress], the TYK-2 inhibitor deucravacitinib (with positive phase II data in psoriasis^63^ and psoriatic arthritis) and an inhibitor of the mitogen-activated protein (MAP) kinase-activated protein kinase-2 (MK2) pathway CC-99677 currently being investigated in axSpA (ClinicalTrials.gov ID: NCT04947579). The efficacy of JAK inhibitors in axSpA suggests that additional cytokines (other than IL-17A/F and TNF) play a role in axSpA that could be targeted using monoclonal antibodies or nanobodies. While IL-23 and IL-6 receptors are well-known activators of JAKs, antibody-mediated neutralization of these cytokines was not effective in axSpA patients, neither IL-17A/F nor TNF signal through the JAK/signal transducer and activator of transcription (STAT) signalling pathway. One potential candidate is granulocyte-macrophage colony-stimulating factor (GM-CSF), which does signal through the JAK/STAT pathway, and an anti-GM-CSF antibody is currently being tested in a clinical trial in axSpA (ClinicalTrials.gov ID: NCT03622658).

The inhibition of structural damage in the spine (formation of syndesmophytes and ankylosis, often referred to as radiographic progression) is a desirable treatment outcome in axSpA. Current knowledge suggests that inflammation is the first step in the process leading to structural damage and pathological new bone formation.^5^ Early and effective inhibition of inflammation should therefore prevent radiographic progression. Studies in that regard are ongoing and include studies investigating a potential immediate inhibitory effect of NSADs (celecoxib, a cyclooxygenase-2 selective inhibitor) added to a TNF inhibitor and of the IL-17A inhibitor secukinumab (compared with a TNF inhibitor) on radiographic spinal progression in high-risk patients with AS.^64,65^ Positive results of these studies (expected 2022) could affect the treatment approach for patients presenting with known risk factors for rapid and extensive structural damage development in the spine – early syndesmophytes, elevated C-reactive protein (CRP). The current approach to prevent syndesmophyte formation focuses on early diagnosis and suppression of inflammation. How much of a need there is for drugs that specifically target new bone formation beyond inhibition of inflammation is an open question. Potential benefits need to be weighed against risks as well as added cost. Long-term observational studies have demonstrated large individual differences in radiographic progression.^66,67^ Subsets of patients may indeed benefit from additional therapy. The development of more precise biomarkers to identify patients at risk seems important, however, both to document the efficacy of such drugs in clinical trials and to limit treatment to those patients who will benefit from the intervention.

Peripheral SpA is another important and evolving indication within the SpA family. Peripheral SpA is a term covering patients with clinical and lab characteristics typical for SpA but without axial manifestations. The so-called chronic reactive arthritis, arthritis associated with inflammatory bowel disease, some forms of psoriatic arthritis (SpA-like) as well as SpA with peripheral manifestations, which cannot be classified otherwise, belong to this entity. Currently, there are no approved treatment options (especially no b- or tsDMARDs) for patients presenting with active pSpA that cannot be diagnosed/classified as axSpA or as psoriatic arthritis. Several years ago, a phase III study with adalimumab in non-psoriatic pSpA patients provided positive results,^68^ which, however, did not result in an attempt to obtain approval for these indications. The high efficacy of TNF inhibition in this disease has also been shown in an investigator-initiated study with golimumab.^69^ More recently, a phase III study with secukinumab in pSpA has been announced (ClinicalTrials.gov ID: NCT05206591). We expect that within the next decade, several effective anti-inflammatory treatment options will become available for SpA patients presenting with peripheral manifestations.

In SpA, as in any chronic and potentially severe and disabling disorder, there is a wish to achieve a cure or at least long-term (and ideally drug-free) remission. As in many other chronic immune-mediated disorders, the disease cannot be cured because of complex, profound and still not entirely understood immunological disease mechanisms. Currently available drugs (NSAIDs, TNF, IL-17A and JAK inhibitors) can induce remission (defined as absence of clinical and lab evidence of disease activity)^70^ in a certain proportion of patients – approximately 20–25% of patients with long-standing disease that can go up to 60% in patients at the early disease stage.^71^ Treatment discontinuation, however, results in a disease flare in the majority of patients.^54,72,73^ To date, no clinically relevant predictors of disease flare/sustained remission are known. Therefore, there is a need for new treatment options that can re-establish immune homeostasis and long-term drug-free remission.

Haematopoietic stem cell transplantation was described in a few case reports as a procedure that might be associated with the induction of remission in axSpA.^74^ The procedure is thought to result in an ‘immune system reset’ that leads to remission of inflammation. The costs and risks associated with autologous or allogenic stem cell transplantation are very high, which makes it unlikely that stem cell transplantation will play any significant role in treating a non-fatal disease like SpA. Mesenchymal stem cells, which have a potential to act as immunoregulators, might be an alternative to a stem cell transplantation, but currently there are no clinical studies supporting their use in SpA.

The progress in high throughput technologies in biomedical research and bioinformatics has renewed interest in the role of CD8+ and CD4+ cells in the pathophysiology of SpA-inflammation^75,76^ and indicated a potential for the identification of ‘disease-relevant’ T cells,^77^ especially in HLA-B27-positive subjects.^78,79^ These findings might lead to new treatment approaches, for example, by depletion of ‘disease-specific’ expanded T cells or by induction of immunological tolerance with exposure to a disease-relevant antigen (which are currently unknown but could potentially be identified using approaches successfully applied in psoriasis).^80^

**Figure 1.**
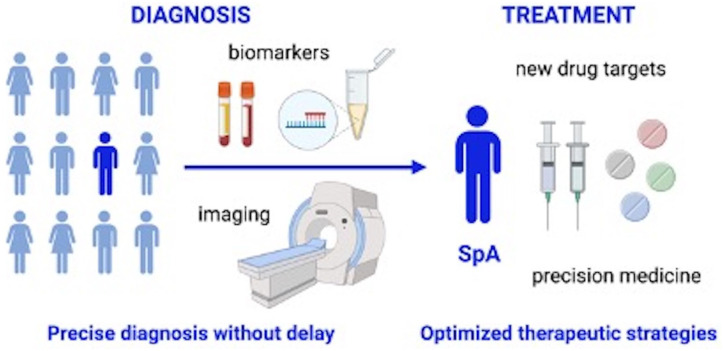
The future of SpA.

In summary, in the treatment of SpA, we can expect an increasing number of novel drugs, most of which will be directed against intracellular targets. We hope to see more strategy trials shaping treatment pathways in SpA and accommodating principals of precision medicine. There will be approved treatment options not only for axial but also for peripheral SpA. Finally, we hope to intervene not only at the inflammation level but also at the level of the underlying immunological processes which may increase the probability of achieving long-term remission if not a cure (Figure 1).

We anticipate shortening of diagnostic delay and improvement of the diagnosis accuracy by establishing specific referral strategies, imaging tools and lab biomarkers for axial spondyloarthritis. We expect an expansion of the available treatment modalities and optimization of treatment strategies with implementation of precision medicine approaches. The figure was generated with BioRender.

## Declarations

**Ethics approval and consent to participate:** Not applicable.

**Consent for publication:** Not applicable.

**Availability of data and materials:** Not applicable.
